# Potential for Ammonia Recapture by Farm Woodlands: Design and Application of a New Experimental Facility

**DOI:** 10.1100/tsw.2001.338

**Published:** 2001-11-30

**Authors:** Mark R. Theobald, Mark C. Milford, Mark K.J. Hargreaves, Mark L.J. Sheppard, Mark E. Nemitz, Mark Y.S. Tang, Mark V. R. Phillips, Mark R. Sneath, Mark L. McCartney, Mark F. J. Harvey, Mark I. D. Leith, Mark J. N. Cape, Mark D. Fowler, Mark M. A. Sutton

**Affiliations:** Centre for Ecology and Hydrology, Edinburgh, UK

**Keywords:** abatement, ammonia, emission, NH_3_, reduction, SF_6_, shelterbelt, sulphur-hexafluoride, throughfall, tracer, trees, woodland

## Abstract

There has been increasing pressure on farmers in Europe to reduce the emissions of ammonia from their land. Due to the current financial climate in which farmers have to operate, it is important to identify ammonia control measures that can be adopted with minimum cost. The planting of trees around farmland and buildings has been identified as a potentially effective and low-cost measure to enhance ammonia recapture at a farm level and reduce long-range atmospheric transport. This work assesses experimentally what fraction of ammonia farm woodlands could potentially remove from the atmosphere. We constructed an experimental facility in southern Scotland to simulate a woodland shelterbelt planted in proximity to a small poultry unit. By measuring horizontal and vertical ammonia concentration profiles within the woodland, and comparing this to the concentration of an inert tracer (SF6) we estimate the depletion of ammonia due to dry deposition to the woodland canopy. Together with measurements of mean ammonia concentrations and throughfall fluxes of nitrogen, this information is used to provide a first estimate of the fraction of emitted ammonia that is recaptured by the woodland canopy. Analysis of these data give a lower limit of recapture of emitted ammonia, at the experimental facility, of 3%. By careful design of shelterbelt woodlands this figure could be significantly higher.

## INTRODUCTION

The deposition of atmospheric ammonia ($NH_3$) is a particular problem of rural areas because the majority of ammonia emissions are a result of agricultural practices[1]. Ammonia has a rather short atmospheric lifetime, estimated at 1 to 3 h[2], before being either converted to ammonium aerosol, washed out by rain, or dry deposited. Since most of the deposition occurs within a few kilometres of the source, problems arise where there is a sensitive seminatural habitat near to an intensively farmed area. This deposition of nitrogen can cause eutrophication of the ecosystem and acidification of the soil[3]. On a species level, this can lead to out-competition of oligotrophic plants by nitrophilous species resulting in a loss of biodiversity[4,5]. One obvious way of tackling these problems is to reduce the amount of ammonia emitted from the farm system. Given the costs of abatement techniques[6], there is a need to explore novel, low-cost methods to decrease ammonia emissions.

Previous studies have shown that a higher rate of ammonia deposition occurs onto woodland canopies compared with other vegetation types[2,4,7,8]. This is because there is a higher deposition velocity for ammonia on to a woodland canopy compared with that of an aerodynamically smoother surface (e.g., grassland) due to the greater turbulence and leaf surface area of the canopy[8]. Another benefit of the turbulence created by the woodland is a further decrease of the atmospheric ammonia concentration due to the increased mixing of the air, i.e., dilution of the ammonia. As well as being a potentially cost-effective method of abating ammonia emissions, farm woodlands have many other benefits, including acting as a shelter for livestock, providing a source of timber, shielding unsightly buildings, and existing as a habitat for wildlife. In many countries there are grant schemes in place to encourage farmers to plant new woodlands (e.g., for schemes in the U.K. see http://www.forestry.gov.uk/Website/OldSite.nsf/ByUnique/HCOU-4U4J2M), which will increase the cost effectiveness of this abatement strategy.

This paper considers an experimental approach to quantify the proportion of ammonia emission from livestock housing that can potentially be recaptured by a nearby belt of farm woodland. The design of an experimental facility is described to show how several complementary approaches can be used to assess ammonia deposition in a situation where the classical micrometeorologists' assumption of no horizontal concentration gradient clearly does not hold. The paper goes on to report the wide range of results obtained to date and describes how they may be used in subsequent modelling activities. Finally, the paper discusses how the novel design of farm woodlands might be used to maximise the recapture of ammonia.

## EXPERIMENTAL FACILITY

An experimental facility has been established to simulate the ammonia emission from a small poultry unit (24,000 chickens ≡ 2900 kg $NH_3$ $year^{-1}$[9,10]) and to measure the subsequent deposition to a belt of woodland downwind of the emission source. The system was designed using a line source 40 m long at a height of 1 m to provide a simplified representation of the ammonia emission from a side-ventilated poultry building.

### The Woodland

A suitable belt of woodland was found, for the experiment, in southern Scotland within 25 km of the Centre for Ecology and Hydrology. The woodland is perpendicular to the prevailing SW winds and is approximately 150 m long and 60 m wide. This length makes it possible to carry out measurements along a transect away from the source with edge effects being negligible. The canopy is relatively open, allowing air to pass through and not simply over it. It consists mainly of Scots pine with a few birch and spruce trees, and has a maximum canopy height of about 12 m. Upwind of the woodland is a field of short grass, which makes it easier to understand and model the airflow through the woodland. A leaf area density distribution for the woodland is still to be measured.

### Ammonia Release System

A 40-m-long ammonia source was set up, parallel to the woodland, comprising a release manifold and an ammonia release and control system. The manifold consists of a modified fan unit (Rosenberg GMBH, Germany, maximum flow = 3 $m^3$ $min^{-1}$) connected to a 40-m-long polyethene tube (0.26 m in diameter) closed at the end opposite to the fan. Along the length of the tube, 6-mm diameter holes were placed with four around the circumference of the tube at 1-m intervals. The speed of the fan was set so that there was no significant internal pressure loss along the manifold, ensuring a uniform flow of air out of the holes along the tube's length. The manifold was positioned so that its centre was 1 m above the ground and 5 m from a stone wall marking the woodland's edge.

The ammonia release control system is shown in Fig. 1. A cylinder of anhydrous ammonia is connected to the manifold via a mass flow controller (Tylan FC280, maximum flow = 10 l $min^{-1}$ at s.t.p.) and a stainless-steel solenoid valve (KIP 2-way series 3 with neoprene seals and a 12 V operating voltage) using ¼-in.-diameter stainless-steel tubing. The mass flow controller and solenoid valve are also connected to a datalogger (model 21X, Campbell Scientific, Shepshed, U.K.). When the 5-min mean of the wind direction (measured by a Gill ultrasonic anemometer and calculated using vector addition) is within 15° of the normal to the woodland, i.e., the wind is blowing into the trees, and the mean wind speed exceeds 0.2 m $s^{-1}$, the datalogger sends a signal to open the solenoid valve and the ammonia flows into the manifold at a predetermined rate. The release continues until the 5-min mean of the wind direction falls outside 15° from the normal or the wind speed decreases to less than 0.2 m $s^{-1}$. In the initial stages of the experiment, the release rate was adjusted to produce ammonia concentrations in the woodland that are typical for downwind of a poultry unit[11], resulting in a release rate of 5.48 g $min^{-1}$. For campaign tracer experiments, an $SF_6$ cylinder (10% $SF_6$ in $N_2$) was connected to the release manifold using ¼-in.-diameter PVC tubing. The flow rate of the $SF_6$ was controlled using a rotameter, with rates between 0.02 and 0.19 g $min^{-1}$ being used for the campaign measurements.

Two safety mechanisms have been incorporated into the release system to prevent undiluted ammonia being released into the air: (1) the solenoid valve is powered from the main supply, therefore if power fails at the facility the ammonia release will be stopped; and (2) if the fan stops for any reason or the manifold becomes detached, a pressure sensor measuring the pressure difference between the inside and outside of the manifold detects this. This pressure difference is typically 1.1 kPa, and if it drops below 0.45 kPa the solenoid valve is closed.

### Long-Term Measurements

A range of measurements has been implemented to estimate the proportion of ammonia from the source that is recaptured, as well as providing data that could be used to validate dispersion

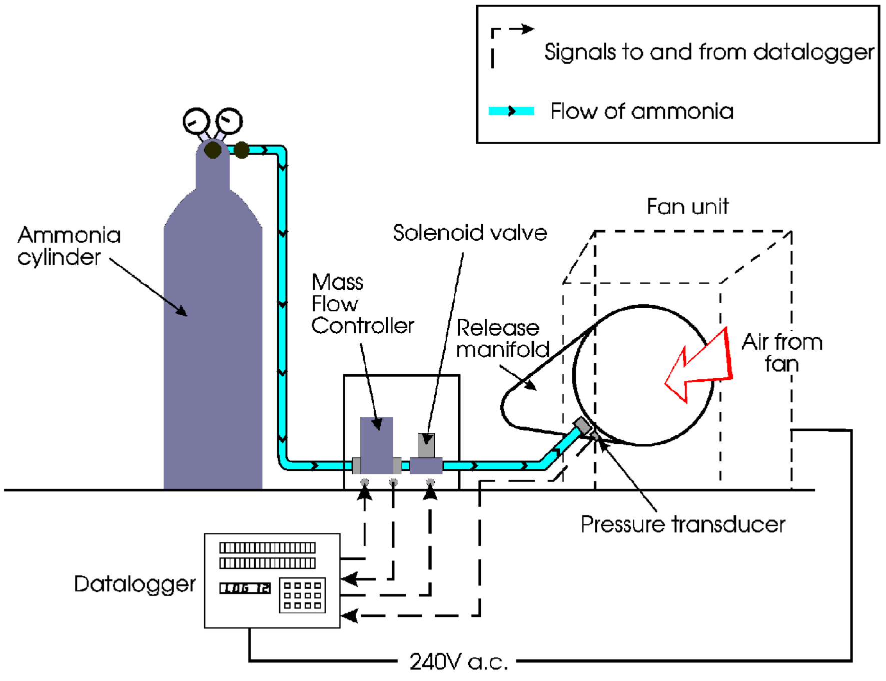

**FIGURE 1.** Diagram of the ammonia release and control system.

models. The measurements can be divided into two classes; long-term and campaign measurements. The long-term measurements provide an estimate of recapture on a seasonal scale with sampling times integrated over a few weeks. Conversely, the campaign measurements give an estimate of recapture with a time resolution of a few minutes.

## Throughfall Measurements

Throughfall collectors were placed at nine locations in the woodland along a transect away from the source as shown in Fig. 2. Three extra collectors are located in a part of the woodland that is outside the fumigation zones (approximately 40 m from the upwind woodland edge and 40 m NW of the line running SW–NE through the centre of the source) to act as control samples. The method of collection was a sloping plastic gutter situated under the woodland canopy, 0.3 m above ground, connected to a black plastic tank. A biocide (thymol) was present in the tank, suspended as a solid in a mesh thimble, to provide a saturated solution to prevent sample decomposition. The throughfall water was sampled and emptied at intervals of 2 to 8 weeks (depending on the volume of rainfall for the period). Throughfall was collected for ten periods spanning a total time of 237 days. The volume of water in the tank was recorded and the pH, electrical conductance, and ammonium ($NH_4^+$) ion concentration of the sample were measured. For the pH measurement the sample was mixed with 1 M KCl solution, giving a solution of constant high ionic strength, which was then passed through a flow-through cell and the pH measured using a Whatman microelectrode at room temperature. Solutions of dilute sulphuric acid were used to calibrate the electrode over the pH range 4–6. The conductivity measurements were made using a continuous flow system with a Wescan detector for ion chromotography at 35°C. The analysis for $NH_4^+$ concentration uses a flow-injection analysis system[12].

## Rainfall Measurements

Three rainfall collectors were located NW of the woodland. These provided an estimate of the composition of the rainfall that falls onto the woodland canopy. The collectors consisted of a 0.2-m diameter polyethene funnel attached to a 2.5-l black polyethene bottle. The top of the funnel was at a height of 1.5 m. Samples were preserved in situ with thymol as for the throughfall samples. Measurements of pH, electrical conductance, and $NH_4^+$ ion concentration were made as for the throughfall samples.

## Mean Monthly Ammonia Air Concentrations

Ammonia concentrations were measured at seven locations on a transect away from the source at a height of 1.5 m above the woodland floor. The measurements were made using triplicates of ALPHA samplers[13], which were analysed using the ammonium flow-injection analysis system. A further triplicate set of samplers were located approximately 20 m upwind of the source to provide an estimate of background ammonia concentrations.

## Campaign Measurements

Intensive measurements were carried out during a 4-week campaign during September and October 2000. During this period, measurements of turbulence and ammonia dispersion were made

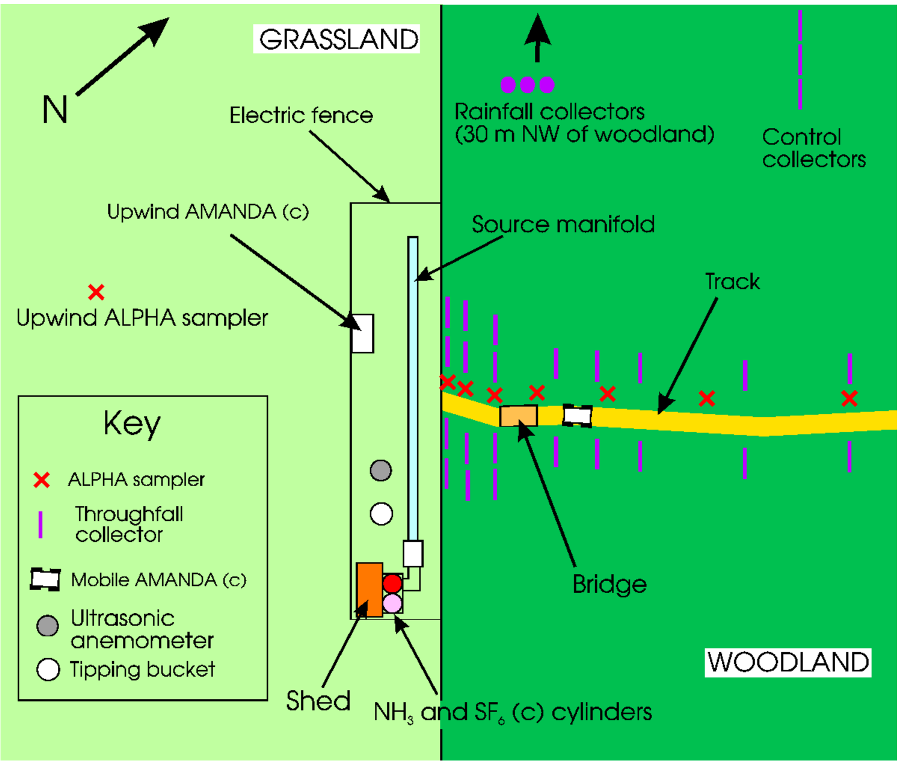

**FIGURE 2.** Layout of the experimental facility (not to scale). (c) indicates presence only during campaign measurements.

within the woodland canopy, together with $SF_6$ dispersion measurements and time-resolved ammonia concentration profiles above the upwind grass field. Within this period the measurements focused on the times when the wind direction was perpendicular to the woodland.

## *Mobile Ammonia Concentration Measurements*

An AMANDA system[14] was used to measure the concentration of ammonia in the air along horizontal and vertical transects. A purpose-built cart was constructed for the denuders, control box, and airpump, which allows the system to be moved whilst running. A sand track was constructed along the woodland floor for the cart to be moved along. A small bridge also had to be constructed so that the cart could be moved over a depression in the woodland floor 3 m wide and 0.5 m deep. Two inlets were used in the AMANDA, and these were both placed at the same height to give duplicate concentration measurements.

To perform the vertical transect measurements, a pump-up-mast (fully extended height: 15 m) was used. Since the AMANDA inlets (including the glass denuder, drive motor, and aluminium box) were too heavy to raise using the mast, 12-m-long tubes (½-in. outside diameter polyethene) were attached to the inlets and these were raised. Measurements made using an AMANDA with such inlets have shown that, in dry conditions, conditioned tubes do not adsorb ammonia[15]. The airflows along the inlets (approximately 25 l $min^{-1}$) should ensure that the tubes were dry during the measurements.

## *$SF_6$ Measurements*

By inserting a Teflon T-piece into the AMANDA inlet tube, it was possible to subsample from the airflow into 3-l polyvinylfluoride bags for analysis of $SF_6$ concentration. This was done on both of the AMANDA inlets to allow duplicate bag sampling. The bags were filled using the “lung principle”, which involved filling the bags within a pre-evacuated barrel. The pressure difference between the outside and inside of the bag caused air from the AMANDA inlet to be sucked into the bag. This was done so that the sampled air did not come into contact with any internal pump surfaces. Two inlets on the barrel made it possible to fill two bags at the same time. The bags were stored at 4°C (to minimise losses from the bag) until the samples were analysed for $SF_6$ content using a gas chromatography (GC) system (Chrompack CP9001, with a 50-m PoraPLOT Q fused silica column at an oven temperature of 50°C). An electron capture detector at 375°C was used as the detector, with an ECD-grade nitrogen carrier gas.

The protocol for the ammonia and $SF_6$ sampling was as follows: after the AMANDA inlets had been moved into position (either by moving the cart for the horizontal profiles or by raising the mast for the vertical profiles), the AMANDA was given time for the measurement to become stable (usually 15 to 20 min), and then the bags were filled. Two to six samples were collected at each position on the transect, with bag fill-times between 3 and 10 min each. For the analysis, the AMANDA ammonia concentration was averaged over the bag fill-time.

### Within-Canopy Turbulence Measurements

Turbulence measurements were made at the same time as the ammonia and $SF_6$ sampling using a microultrasonic anemometer (50-mm path length, Kaijo-Denki FA-600 with TR-90AH probe). This was mounted close to the AMANDA inlets to measure the turbulence characteristics at the point in the canopy from where the air was being sampled. These measurements were then referenced to those made using the Gill ultrasonic anemometer 8 m upwind of the woodland edge.

### Upwind Ammonia Concentration Measurements

During the intensive campaign a second AMANDA was located 5 m upwind of the source to measure the vertical ammonia concentration profile above the upwind grass field. Three inlets were used at heights of 0.39, 0.74, and 1.97 m.

### Calculation of Ammonia Depletion Due to Recapture Using the Tracer-Ratio Method

One of the techniques used for quantifying the ammonia recapture by the woodland was the tracer-ratio method. In this method a tracer gas, $SF_6$, is released from the source at the same time as ammonia and the air concentrations of the two gases are measured at various locations within the woodland. A similar technique has been used by previous studies to estimate the source strengths of various pollutants such as methane[16] and isoprene[17], and have produced emission estimates within 15% of the known values. In these studies, it was assumed that the ratio between the source strengths of the tracer and pollutant is the same as the ratio of the measured air concentrations of the two gases, provided that the tracer and pollutant plumes were well mixed. For ammonia and $SF_6$ these ratios are assumed not to be the same with distance from the source, due to the nature of the two gases. $SF_6$ is an extremely stable gas, has an estimated atmospheric lifetime of 800 to 3200 years[18,19], and has a low solubility in water[20]. Ammonia, in contrast, is very reactive, giving an atmospheric lifetime of a few hours[2], and is very soluble in water. Due to these differences, it is assumed that there will be minimal deposition of $SF_6$ to the canopy compared with ammonia, which will result in a decrease of the ratio of the air concentration of ammonia to that of $SF_6$ with distance away from the source. It is also assumed that differences in dispersion of the two gases due to molecular dispersion are negligible at wind speeds greater than 0.2 m $s^{-1}$.

An estimate of the depletion of ammonia due to recapture can be obtained by considering the ratio, $r$, where:

$$r = \frac{\left(\chi^{e}_{NH_3} - \chi^{bg}_{NH_3}\right) / E_{NH_3}}{\left(\chi^{e}_{SF_6} - \chi^{bg}_{SF_6}\right) / E_{SF_6}} \quad (1)$$

Where $\chi$ is concentration (μg $m^{-3}$), E is emission (μg $s^{-1}$), $^{e}$ denotes the measured concentration, and $^{bg}$ denotes the background concentration in the air.

## RESULTS

### Ammonia Air Concentration Measurements

Figure 3 shows the means of the triplicate ALPHA sampler measurements along the transect for two of the exposure periods: (a) a 24-day period in Autumn 2000 and (b) a 40-day period in Spring 2001. The total ammonia release for the first period was 0.22 $m^3$, and for the second period it was 11.25 $m^3$. The ammonia release rate was changed from 0.92 to 5.48 g $min^{-1}$ between these two periods to produce ammonia concentrations closer to those occurring in the vicinity of a small poultry unit[11]. The measurements show an approximately logarithmic decrease in ammonia concentration away from the ammonia source. At the small release rate used, the concentration of ammonia near the downwind edge of the woodland is similar to that measured upwind of the source indicating the effect of the woodland in depleting ammonia concentrations by dispersion and deposition. By contrast, at the larger release rate the concentration at the downwind edge of the woodland is still >4 times the background concentration. The profiles may be normalised by dividing all of the measurements by the mean ammonia release rate (μg $s^{-1}$) during the respective sampling period. Fig. 4 shows the concentrations for all of the sampling periods normalised in this way with the background concentration subtracted from all samples. Most of the measured profiles show the measurement at 26.8 m from the source to be slightly above the smooth decay curve formed by the other points. Since this is present on most of the profiles, it is very likely that this is a real effect due to a feature of the woodland. This has yet to be confirmed, but may be related to the topography of the woodland floor or to variations in the density of the canopy above the sampling location.

### Throughfall Measurements

Figure 5 shows the effect of the ammonia release on the throughfall flux of $NH_4^+$ along the transect for three example sampling periods. This was calculated by subtracting the mean mass of $NH_4^+$ in the control collectors from that in each of the collectors in the fumigated part of the woodland and converting this into units of kg/ha using the area of the gutter (0.44 $m^2$). Each value plotted is the mean of all of the collectors at that distance from the source. All three profiles show a decrease in $NH_4^+$ throughfall enhancement away from the source. This would be expected, since the ammonia air concentration also decreases away from the source. Most of the profiles show an increase from 5.5 to 8.5 m from the source. Since the collectors at 5.5 m from the source are very close to the woodland edge, a significant fraction of the throughfall may blow into the woodland and miss the collectors at the first distance, thus giving a potential underestimate of the deposition of $NH_4^+$. All three of the profiles plotted exhibit a decrease at 26 m from the source. This feature is present in eight of the ten periods. Observations show that the canopy is less dense in this area of the woodland, which may explain a lower deposition than expected. Many of the profiles (eight out of ten) show an increase in deposition towards the downwind edge of the woodland. Two possible explanations for this are that the leaf area density (LAD) above these collectors may be higher than that above the others, or that part of the ammonia

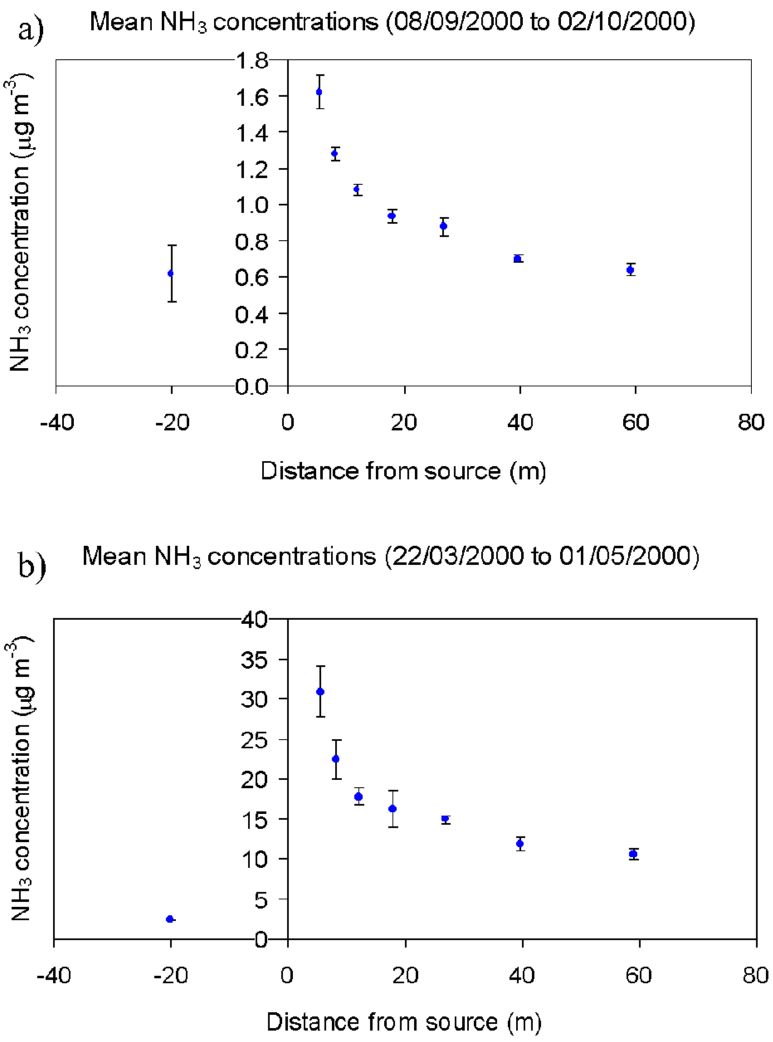

**FIGURE 3.** Example mean ammonia concentration measured by ALPHA samplers, at a height of 1.5 m, as a function of distance from the source. Error bars are ± 1 standard error of triplicate measurements at each location.

plume is passing over the woodland and coming back down towards the downwind edge.

Figure 6 shows the sum of the deposition of $NH_4^+$ over the ten measurement periods (237 days in total) at each measurement location. Two lines are plotted: the actual throughfall flux into the collectors in the fumigated part of the woodland and the same data with the throughfall flux into the control collectors subtracted (5.18 kg $NH_4^+$ $ha^{-1}$). These profiles exhibit many of the characteristics of the individual periods, such as the increase near the woodland edge, the dip at 26 m, and the increase towards the downwind edge of the woodland.

The control-corrected profile can be used to calculate an estimate of the amount of ammonia recaptured during the 237-day period. It is more sensible to do this using a smooth curve fitted to the profile, since the canopy density is variable throughout the woodland. A quadratic was chosen for the fit to the profile since it follows the general form well (see Fig. 6) and is simple to integrate. If $x$ is the distance from the source, the quadratic has the form:

$$\text{Estimated}\ \left(\text{kg NH}_4^+\ \text{ha}^{-1}\right) = 0.0061x^2 - 0.42x + 12.277 \quad (2)$$

Integrating this from 5 to 65 m (the location of the woodland downwind of the source) gives a deposition of 0.041 kg $NH_4^+$ deposited along a line 1 m wide away from the source. By assuming that the deposition profile is constant in the direction perpendicular to the prevailing winds, this deposition can be scaled up to calculate the deposition to the whole fumigated area. Multiplying this by 40 (the length of the source) gives a total deposition of $NH_4^+$ of 1.65 kg, which contains 1.28 kg of nitrogen. The mass released over this period was 58.5 kg $NH_3$–N. Hence an estimated 2.2% of the ammonia was deposited to the branch and leaf surfaces and washed down to the woodland floor. This is not the total recapture, since some of the ammonia is expected to be absorbed by the foliar surfaces. The release rate was increased from 0.92 to 5.48 g $min^{-1}$ at the end of the seventh measurement period. Estimates of deposition (excluding foliar uptake) of 2.9% of the emitted ammonia at the small release rate and 2.0% at the large release rate are obtained if the integration is carried out for these two periods. It is important to note that out of the 80 samples of throughfall collected in the fumigated part of the woodland, 15 overflowed during heavy periods of rain. This will result in an underestimate of the amount of $NH_4^+$

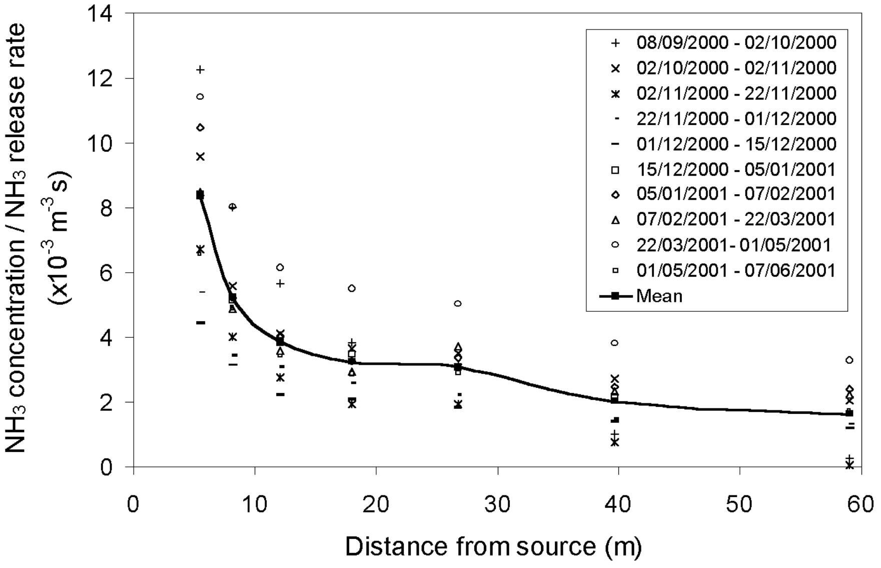

**FIGURE 4.** Normalised mean ammonia concentration profiles measured using the passive (ALPHA) samplers for all sample periods.

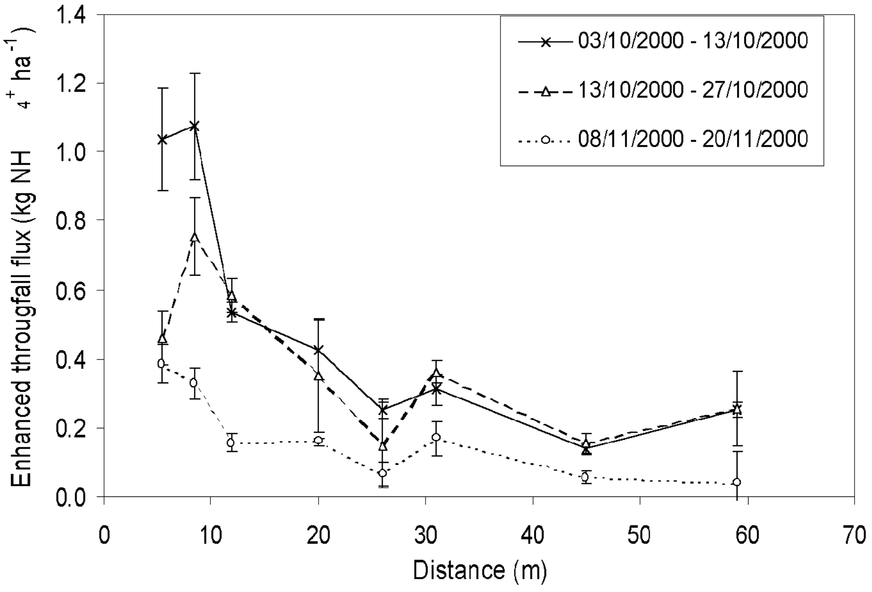

**FIGURE 5.** Enhancement of throughfall $NH_4^+$ fluxes due to the ammonia release along a transect away from the source for three example sample periods. Error bars are ± 1 standard error of the measurements at each distance.

deposited and therefore an underestimate of the proportion of ammonia recaptured.

## Campaign Measurements

### *Ammonia Concentration Profiles*

During the campaign 14 concentration profiles (of both ammonia and $SF_6$) were measured. Fig. 7 shows (a) a horizontal and (b) a vertical profile of ammonia concentrations measured by the AMANDA. (In Fig. 7b the ordinate axis has been chosen to display the height to present the data in a more intuitive way.) The values represent a mean of the measurements for each denuder (A and B) over the time that the AMANDA was at the sampling location. The two denuders agreed very well, and the decrease in air concentration of ammonia away from the source can clearly be seen (Fig. 7a). All of the other horizontal profiles measured at this height show a very similar decrease in concentration. The vertical profile was measured at a distance of 45.2

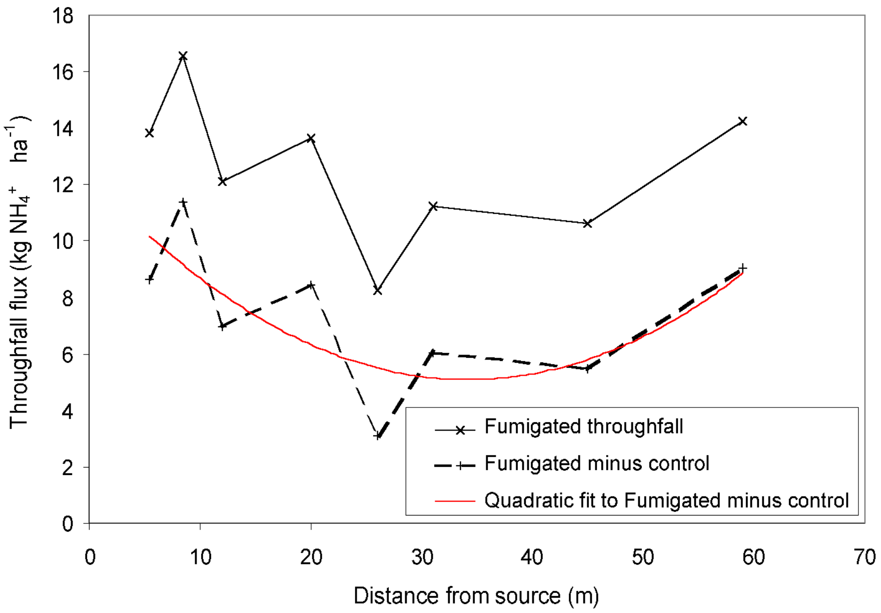

**FIGURE 6.** Total throughfall $NH_4^+$ flux for different distances into the woodland measured over a period of 237 days. The net effect of the ammonia release is shown by the line representing the difference between throughfall under the fumigated trees and under control (unfumigated) trees.

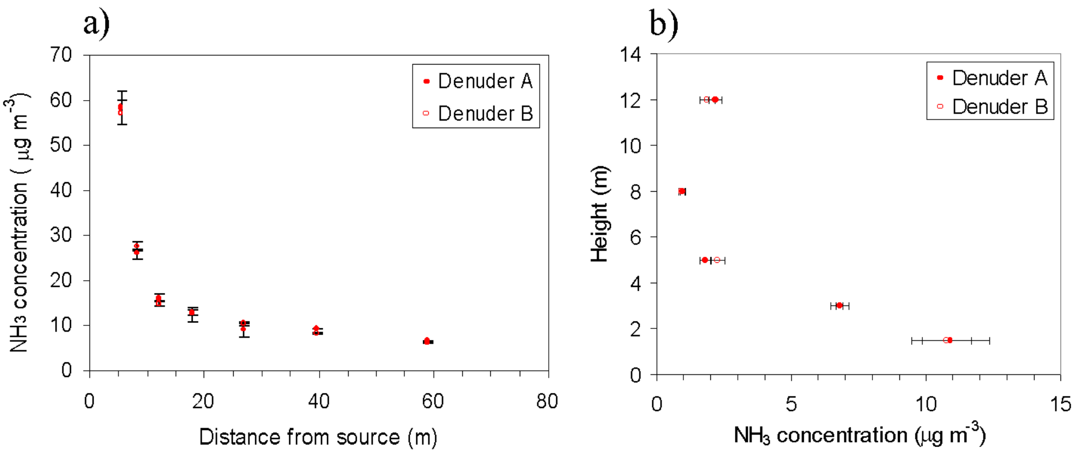

**FIGURE 7.** Ammonia concentration profiles for (a) a horizontal transect at 1.5 m above ground and (b) a vertical transect at 45.2 m from the source. Error bars are ± 1 standard error of the concentrations measured over the period the AMANDA was at each location.

m downwind of the source, and again there is good agreement between the two denuders, and a decrease in ammonia concentrations can be seen, with an increase in height up to 8 m, and then an increase in concentration between 8 and 12 m. Since the average canopy height of the woodland is between 10 and 11 m, the 12-m measurement is above the canopy. The increase in concentration above the canopy may be due to ammonia from the source flowing over the top of the woodland. The other vertical profiles show a similar decrease in concentration up to the canopy height, and, where measurements were made above the canopy, an increase in concentration was again observed.

## $SF_6$ Concentration Profiles

Figure 8 shows the $SF_6$ measurements for the same sample runs as Fig. 7. The measurements for the horizontal transect show a decrease in concentration with distance as for ammonia. The measurements for the vertical transect also show a similar trend

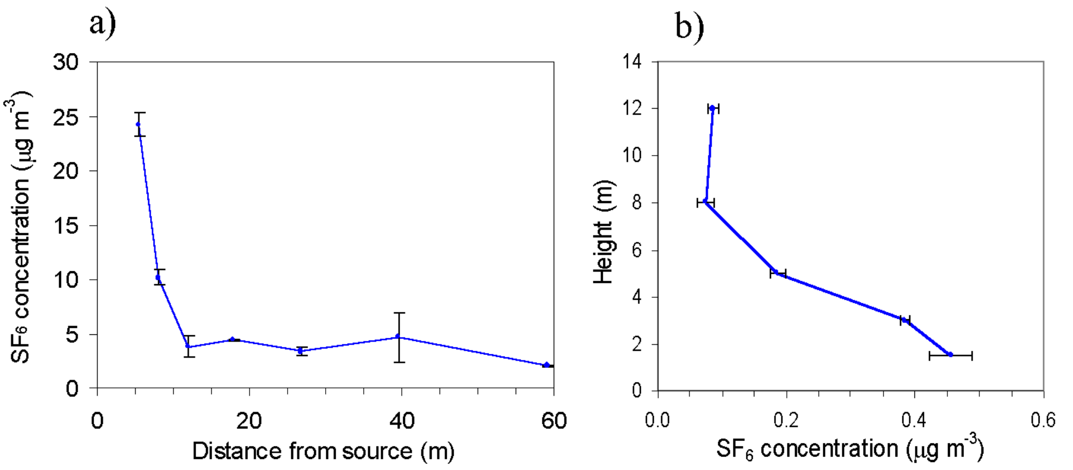

**FIGURE 8.** $SF_6$ concentration profiles along (a) a horizontal transect at 1.5 m above ground and (b) a vertical transect at 45.2 m from source. Error bars are ± 1 standard error of the measurements at each location.

to the ammonia profile, with a decrease in concentration up to 8 m and then a slight increase up to 12 m.

## Tracer Ratios

Fig. 9a shows the ratio (*r*) for the horizontal transect. These values were calculated according to Eq. 1. All but one of the values of *r* are less than unity, suggesting that some preferential recapture of the ammonia has taken place. However, there is no clear decrease in the ratio with distance from the source, which is what would be expected if a greater proportion of the ammonia was preferentially deposited as it moved away from the source. This suggests that little recapture is occurring at this height. Fig. 9b shows the ratio for the vertical profile. The values of *r* form a curve that decreases with height until a minimum is reached at a point within the canopy and then increases above that height. If a quadratic fit to the curve in Fig. 9b is integrated from 0 m to the height of the canopy (10 m) and divided by the canopy height, this gives an estimate of the amount of recapture that has taken place up to the point in the woodland where the measurements were made. This approach gives an estimate of 46 ± 8% cumulative recapture (of the ammonia entering the woodland canopy) at a distance of 45.2 m from the source. This is only a first estimate. A more refined approach will have to take into account weighting of the values according to the measured concentration profile.

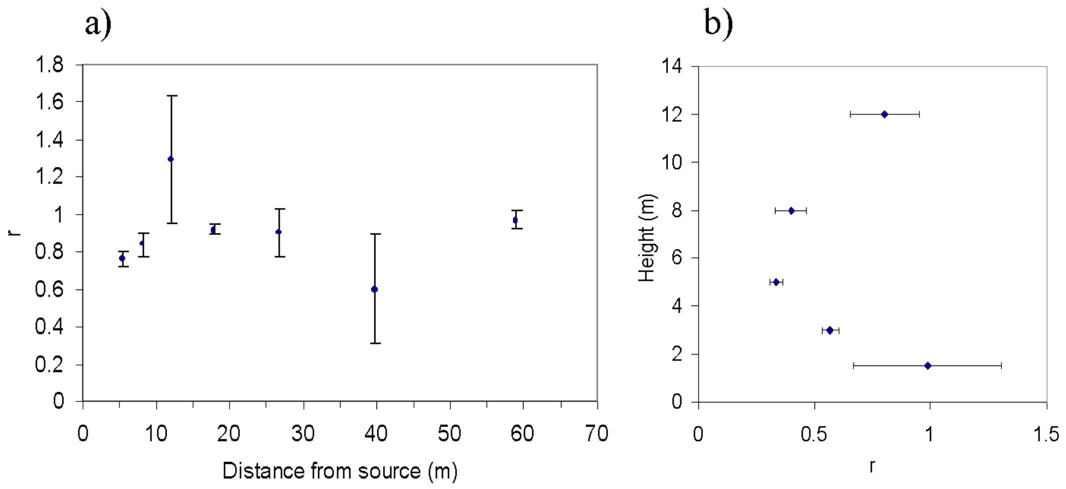

**FIGURE 9.** Values of the tracer ratio (*r*) for (a) a horizontal transect at 1.5 m above ground and (b) a vertical transect at 45.2 m from source. Error bars are ± 1 standard error calculated from standard errors of ammonia and $SF_6$ concentrations and release rates.

## DISCUSSION AND CONCLUSIONS

The throughfall measurements suggest that approximately 3% of the emitted ammonia was recaptured by the woodland. This will be a lower limit for the recapture for several reasons. Firstly, 19% of the samples overflowed during the collection, which will give an underestimate of recapture. By looking at the relationships between the volumes in each of the collectors for each period, it has been estimated that 10% of the rainfall has been lost. The second reason for potential underestimation of recapture is that the area of fumigated woodland is larger than that used in the calculation, which assumes a wind direction perpendicular to the woodland edge, whereas in reality the fumigation can occur in a direction up to 15° off normal. The third reason is that some of the ammonia will be taken up by the foliage of the trees and will therefore not be present in the collected throughfall.

From the tracer-ratio measurements, ammonia recapture of up to 46% of the ammonia entering the woodland is predicted. The concentrations of $SF_6$ measured during the campaign show large uncertainties because many were close to the detection limit of the GC. These low concentrations are the result of the $SF_6$ release rate. The standard errors of the $SF_6$ measurements are smallest for the largest release rate (0.19 g $min^{-1}$). The AMANDA data also showed large uncertainties due to the variation in concentration over time. Both the GC and AMANDA systems can also add a bias to the measurements if the response of the instrument has changed since calibration. This is particularly the case with the AMANDA, which needs to be calibrated weekly, and even then can show significant differences between calibrations. Even though the proportion of ammonia recapture cannot be accurately inferred from these data, the results show that the ratio, *r*, does not vary significantly moving away at a height of 1.5 m from the source, but does vary with height (at 45.2 m from the source), reaching a minimum near the height of maximum LAD.

If farm woodlands are to be used for the purpose of ammonia recapture, they will need to be designed in a way that maximises this effect. The data presented here indicate that the maximum recapture occurs where the LAD is highest, therefore the leaf surface area that the ammonia passes over should be maximised. Using a species of tree that has a large LAD from the ground up to the top of the canopy, but is still sufficiently porous enough to allow the ammonia to pass through, might do this. Scots pine is not ideal for this due to the open understorey. Choice of tree species is therefore a crucial factor, with additional expected differences between evergreen species and deciduous species. Another way of maximising the recapture might be to plant a row of high and dense vegetation at the downwind edge of the woodland. This would act as a barrier to the air passing through the woodland and would force the air and ammonia upwards through the densest part of the canopy and hence the location of the greatest recapture.

The experimental facility reported here will be used in future measurements, which will be refined as a result of the present findings. Experiments will focus on measuring the tracer ratio, *r*, along vertical transects at various distances from the source, since this is the method that produced the most promising data in the study presented here. The effect of having a row of high, dense vegetation at the downwind edge of the woodland may also be simulated by constructing a porous barrier along the edge of the woodland.

The data collected to date will be applied to test and refine a Lagrangian Stochastic model[21] incorporating measurements of turbulence statistics from within the woodland canopy, as well as measurements of LAD. Once the model has been optimised to reproduce the measurements, it may be used to validate the tracer-ratio measurements by simulating the transport of both gases into the woodland. The model could also be used to investigate the effect of woodland design on ammonia recapture.

There are many other considerations when designing the ideal farm woodland. Some of these are practical, such as the suitability of the woodland to provide shelter for livestock. The ideal design for sheltering livestock will be different from that for maximising recapture, since one design is for reducing the wind speed downwind of the woodland (to protect livestock)[22], and the other is for maximising the leaf surface area that the air passes over. Another important consideration is the effect of the ammonium in the throughfall that enters the soil. As a result of nitrification there may be increased nitrate leaching, and the subsequent denitrification of the nitrate may lead to increased $N_2O$ gas emission, both of which are problematic for the environment. These effects will need to be weighed up against the benefits of reducing nitrogen deposition to local sensitive seminatural habitats that may be of conservation priority.

## ACKNOWLEDGEMENTS

We are grateful for the financial support from the U.K. Department for Environment, Food and Rural Affairs (Project No. WA0719) and the U.K. Natural Environment Research Council. We also acknowledge the help and support from David Drury, the farm manager at the site of the experiments.

| | | |
|---|---|---|
| Received: | August | 8, 2001 |
| Revised: | October | 18, 2001 |
| Accepted: | October | 29, 2001 |
| Published: | November | 30, 2001 |
